# The SET Domain Proteins SUVH2 and SUVH9 Are Required for Pol V Occupancy at RNA-Directed DNA Methylation Loci

**DOI:** 10.1371/journal.pgen.1003948

**Published:** 2014-01-22

**Authors:** Zhang-Wei Liu, Chang-Rong Shao, Cui-Jun Zhang, Jin-Xing Zhou, Su-Wei Zhang, Lin Li, She Chen, Huan-Wei Huang, Tao Cai, Xin-Jian He

**Affiliations:** National Institute of Biological Sciences, Beijing, China; Indiana University, Howard Hughes Medical Institute, United States of America

## Abstract

RNA-directed DNA methylation (RdDM) is required for transcriptional silencing of transposons and other DNA repeats in *Arabidopsis thaliana*. Although previous research has demonstrated that the SET domain-containing SU(VAR)3–9 homologs SUVH2 and SUVH9 are involved in the RdDM pathway, the underlying mechanism remains unknown. Our results indicated that SUVH2 and/or SUVH9 not only interact with the chromatin-remodeling complex termed DDR (DMS3, DRD1, and RDM1) but also with the newly characterized complex composed of two conserved Microrchidia (MORC) family proteins, MORC1 and MORC6. The effect of *suvh2suvh9* on Pol IV-dependent siRNA accumulation and DNA methylation is comparable to that of the Pol V mutant *nrpe1* and the DDR complex mutant *dms3*, suggesting that SUVH2 and SUVH9 are functionally associated with RdDM. Our CHIP assay demonstrated that SUVH2 and SUVH9 are required for the occupancy of Pol V at RdDM loci and facilitate the production of Pol V-dependent noncoding RNAs. Moreover, SUVH2 and SUVH9 are also involved in the occupancy of DMS3 at RdDM loci. The putative catalytic active site in the SET domain of SUVH2 is dispensable for the function of SUVH2 in RdDM and H3K9 dimethylation. We propose that SUVH2 and SUVH9 bind to methylated DNA and facilitate the recruitment of Pol V to RdDM loci by associating with the DDR complex and the MORC complex.

## Author Summary

Small RNA-induced transcriptional silencing at transposable elements and other DNA repeats is an evolutionarily conserved mechanism in plants, fungi, and animals. In *Arabidopsis thaliana*, an RNA-directed DNA methylation pathway is involved in transcriptional silencing. Noncoding RNAs produced by the plant-specific DNA-dependent RNA polymerase V are required for RNA-directed DNA methylation. A chromatin-remodeling complex was previously demonstrated to be required for the occupancy of DNA-dependent RNA polymerase V at RNA-directed DNA methylation loci. Our results suggest that two putative histone methyltransferases are inactive in their enzymatic activity and act as adaptor proteins to facilitate the recruitment of DNA-dependent RNA polymerase V to chromatin by associating with the chromatin-remodeling complex. In combination with previous studies, we propose that the inactive histone methyltransferases bind to methylated DNA, thereby linking DNA methylation to Pol V transcription at RNA-directed DNA methylation loci.

## Introduction

DNA methylation is an important epigenetic modification that contributes to transposable element silencing, genome stability, and gene regulation in plants and animals [1]–[3]. In *Arabidopsis thaliana*, DNA methylation can be established through a well-characterized RNA-directed DNA methylation (RdDM) pathway [1], [2]. The RdDM pathway is involved in the silencing of transposable elements and DNA repeats [1], [2], the stability of the genome [4], [5], the development of gametophytes and embryos [6]–[8], and the inheritance of stress-induced transcriptional silencing [9].

In the RdDM pathway, the multi-subunit DNA-dependent RNA polymerase IV (Pol IV) collaborates with RNA-DEPENDENT RNA POLYMERASE 2 (RDR2) to produce double-stranded RNAs, which are subsequently cleaved into 24-nt siRNAs (small interfering RNAs) by DICER-LIKE 3 (DCL3) [10]–[13]. The recruitment of Pol IV to chromatin requires SAWADEE HOMEODOMAIN HOMOLOG 1 (SHH1)/DNA-BINDING TRANSCRIPTION FACTOR 1 (DTF1) [13], [14]. Pol IV-dependent siRNAs are loaded onto ARGONAUTE 4 (AGO4) in the cytoplasm and then transported into the nucleus to complete the assembly of the RdDM effector complex [15]. The multi-subunit DNA-dependent RNA polymerase V (Pol V) produces long noncoding RNAs, which act as scaffold RNAs for the assembly of the RdDM effector complex [16]–[18]. The DDR complex, which is composed of DEFECTIVE IN MERISTEM SILENCING 3 (DMS3), DEFECTIVE IN RNA-DIRECTED DNA METHYLATION 1 (DRD1), and RNA-DIRECTED DNA MEETHYLATION 1 (RDM1), is required for the recruitment of Pol V to RdDM loci and for the accumulation of Pol V-produced noncoding RNAs [17]–[23]. RNA-DIRECTED DNA METHYLATION 4 (RDM4)/DEFECTIVE IN MERISTEM SILENCING 4 (DMS4), an IWR1-like protein, is likely to act as a general transcription factor that is shared by Pol II, Pol IV, and Pol V [24]–[26]. KOW-CONTAINING TRANSCRIPTION FACTOR 1 (KTF1)/SUPPRESSOR OF TY INSERTION 5-LIKE (SPT5L) can bind to Pol V-produced noncoding RNAs and is recruited to chromatin in parallel with AGO4 [27]–[29]. INVOLVED IN DE NOVO 2 (IDN2) and IDN2 PARALOG 1 and 2 (IDP1 and IDP2) can form a complex required for RdDM [30]–[32]. IDN2 physically associates with the SWI/SNF chromatin-remodeling complex, which is required for noncoding RNA-mediated transcriptional silencing [33]. DOMAIN REARRANGED METHYLTRANSFERASE 2 (DRM2) is the main DNA methyltransferase that catalyzes DNA methylation at RdDM loci [34], [35]. MORC1 and MORC6, two members of the conserved Microrchidia (MORC) adenosine triphosphatase (ATPase) family, were thought to be required for decondensation of pericentromeric heterochromatin in a DNA methylation-independent manner [36]. However, another two reports indicated that MORC6/DEFECTIVE IN MERISTEM SILENCING 11 (DMS11) is involved in RNA-directed DNA methylation and transcriptional silencing [37], [38]. Further study is required to clarify the functional mechanism of the MORC family proteins in transcriptional silencing.

In *Arabidopsis*, H3K9 methylation is catalyzed by the conserved SU(VAR)3–9 homologs (SUVHs) and related proteins (SUVRs) [2], [39]. The SUVHs contain a catalytic SET domain and an N-terminal SRA (SET- or RING-associated) domain [39]. The active histone H3K9 methyltransferases SUVH4/KRYPTONITE (KYP), SUVH5, and SUVH6 are not only required for H3K9 dimethylation (H3K9me2) but also for DNA methylation [40], [41]. The connection between DNA methylation and histone H3K9me2 is dependent on the SRA domain in the SUVHs that directly bind to methylated DNA [42], [43]. CHROMOMETHYLASE 3 (CMT3), a plant-specific DNA methyltransferase, can directly bind to H3K9me2-containing nucleosomes and connect the histone H3K9me2 with DNA methylation at CHG sites [44]. At RdDM loci, canonical RdDM components including Pol IV, Pol V, and AGO4 are required for both DNA methylation and histone H3K9me2 [17], [45], [46]. Previous reports demonstrated that SUVH2 and SUVH9 are involved in RNA-directed DNA methylation and transcriptional silencing [47], [48], but the underlying mechanism is unknown.

In this study, we found that SUVH2 and SUVH9 associate with the DDR complex and the newly characterized MORC1-MORC6 complex and act as adaptor proteins to mediate Pol V occupancy at RdDM loci. The conserved catalytic active site of SUVH2 is not required for the function of SUVH2 in RdDM and histone H3K9me2. The results revealed the functional mechanism of SUVH2 and SUVH9 in the RdDM pathway.

## Results

### SUVH2 and SUVH9 associate with DDR components and MORC family proteins *in vivo*

**Figure 1:**
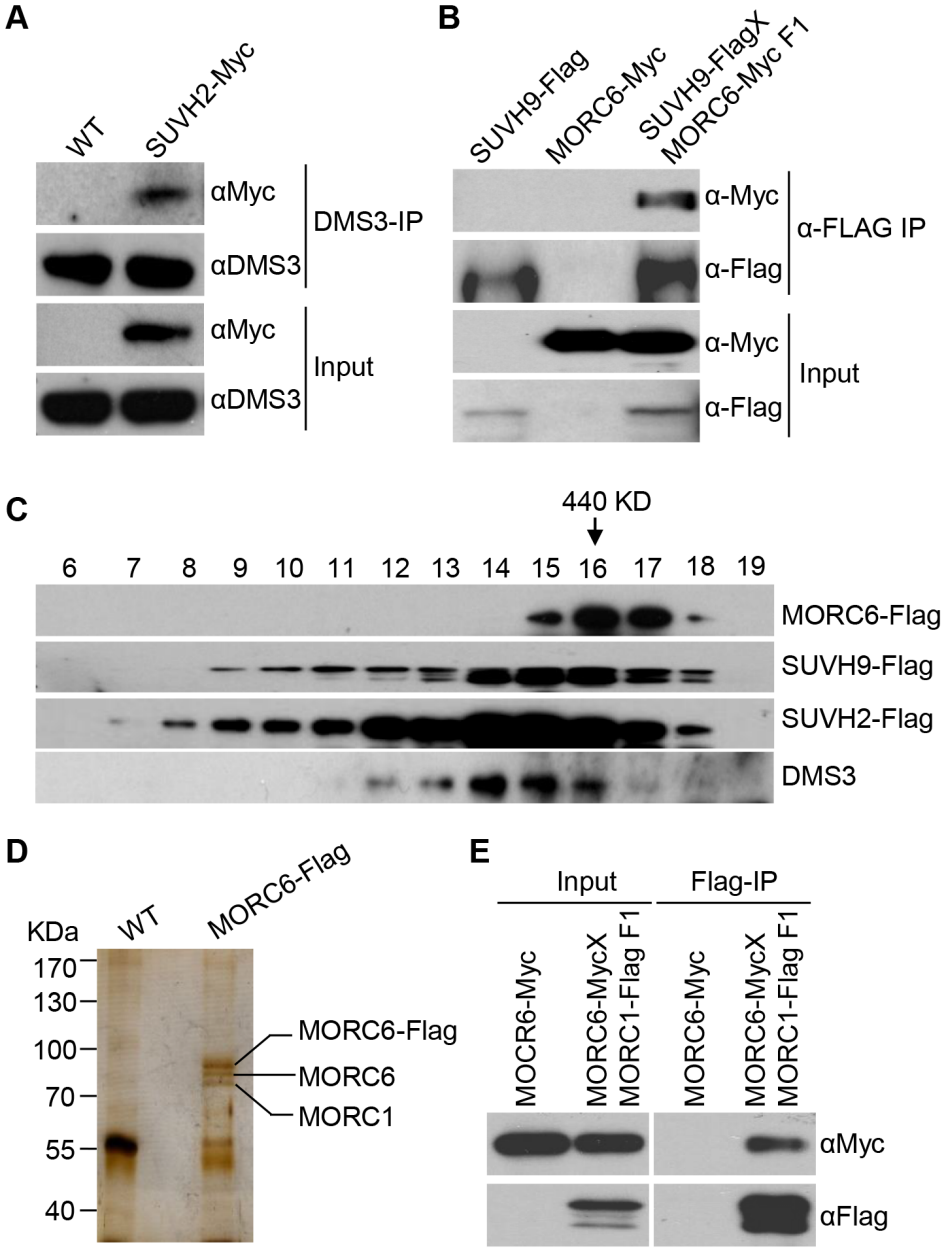
Detection of the protein interaction by co-IP and gel filtration analyses. (**A**) Detection of the interaction between SUVH2-3xMyc and DMS3 by co-IP. The protein extract of *SUVH2-3xMyc* transgenic plants was immunoprecipitated by anti-Myc antibody. The precipitate was subjected to Western blotting with the antibodies against the Myc epitope and endogenous DMS3. (B) The interaction between SUVH9-3xFlag and MORC6-3xMyc as determined by co-IP. The F1 offspring generated from the cross between *SUVH9-3xFlag* and *MORC6-3xMyc* transgenic plants were used for co-IP. (C) The elution profile of MORC6-3xFlag, SUVH2-3xMyc, SUVH9-3xFlag, and DMS3 as determined by gel filtration. Antibodies specific for the Flag epitope and endogenous DMS3 were used for Western blotting. The arrow indicates the fraction of a 440-KDa standard protein. (D) The protein extracts from the wild type and MORC6-3xFlag transgenic plants were affinity purified by anti-Flag antibody. The purified proteins were run on an SDS-PAGE gel and subjected to silver staining. The three separated bands at ∼70–100 KDa were cut out for mass spectrometric analysis. (E) The interaction between MORC1 and MORC6 as determined by co-IP.

SUVH2 and SUVH9 were previously demonstrated to be involved in RNA-directed DNA methylation [47], [48], but the underlying mechanism is unclear. To understand the role of SUVH2 and SUVH9 in RdDM, we generated the native promoter-driven *SUVH2-3xMyc* and *SUVH9-3xFlag* transgenic plants. The stably expressed *SUVH2-3xMyc* and *SUVH9-3xFlag* transgenic lines were used for affinity purification of the proteins that associate with SUVH2 and SUVH9. Mass spectrometric assay revealed that SUVH2 and SUVH9 were the most abundant proteins in affinity purification of SUVH2-3xMyc and SUVH9-3xFlag, respectively (Table 1). Moreover, two RdDM components DMS3 and DRD1 were identified in affinity purification of SUVH2-3xMyc, whereas MORC6/DMS11 and DMS3 were identified in affinity purification of SUVH9-3xFlag (Table 1). The presence of RdDM components DMS3 and DRD1 in affinity purification of SUVH2-3xMyc and SUVH9-3xFlag suggests that SUVH2 and SUVH9 are involved in RdDM by physically associating with RdDM components. MORC6/DMS11 was recently demonstrated to be involved in RNA-directed DNA methylation and transcriptional silencing [37], [38]. The finding of MORC6 in affinity purification of SUVH9-3xMyc suggests that MORC6 interacts with SUVH9. We carried out affinity purification of MORC6-3xFlag in *MORC6-3xFlag* transgenic plants and identified SUVH9 by mass spectrometric assay, confirming that SUVH9 can interact with MORC6 *in vivo* (Table 1). To confirm the interaction between SUVH2 and DMS3, we performed co-immunoprecipitation (co-IP) in *SUVH2-3xMyc* transgenic plants using either anti-Myc antibody or anti-DMS3 antibody. The results indicated that SUVH2-3xMyc and DMS3 were co-precipitated not only by anti-DMS3 antibody but also by anti-Myc antibody (Figure 1A; Figure S1A). The interaction between SUVH9 and MORC6 was also demonstrated by co-IP in the transgenic plants harboring both *SUVH9-Flag* and *MORC6-Myc* transgenes (Figure 1B). We therefore concluded that SUVH2 and/or SUVH9 can physically associate with the canonical RdDM components *in vivo*. However, we could not detect the interaction between SUVH9 and DMS3 by co-IP (Figure S1B, S1C), suggesting that the interaction is either weak or highly locus-specific.

**Table 1:** List of affinity co-purified proteins of SUVH2-Myc, SUVH9-Flag, and MORC6-Flag.

Accession number	Annotation	Protein	Mascot score	MW (Kda)	Spectra counts	Unique peptides
**SUVH2 co-purified proteins**						
IPI00548715	AT2G33290	SUVH2	4009	72801	103	38
IPI00518285	AT3G49250	DMS3	158	46738	4	4
IPI00539494	AT2G16390	DRD1	64	100166	1	1
**SUVH9 co-purified proteins**						
IPI00529277	AT4G13460	SUVH9	7419	72128	187	31
IPI00529245	AT1G19100	MORC6/DMS11	52	74129	1	1
IPI00518285	AT3G49250	DMS3	37	46738	1	1
**MORC6/DMS11 co-purified proteins**						
IPI00529245	AT1G19100	MORC6/DMS11	12402	74129	252	50
IPI00522658	AT4G36290	MORC1/CRT1	4925	70763	114	34
IPI00530171	AT4G36280	MORC2	1236	69934	38	21
IPI00517635	AT4G34430	CHB3/SWI3D	148	107858	2	2
IPI00529277	AT4G13460	SUVH9	132	72128	3	3

We carried out gel filtration to determine whether SUVH2, SUVH9, and related RdDM components act as protein complexes *in vivo*. For gel filtration, the protein extracts from the wild-type plants and *SUVH2-3xMyc*, *SUVH9-3xFlag*, and *MORC6-3xFlag* transgenic plants were separately eluted and then subjected to Western blotting (Figure 1C). The elution profile of these protein extracts revealed that each protein (SUVH2-3xMyc, SUVH9-3xFlag, and MORC6-3xFlag) was eluted in the form of a high-molecular-weight protein complex rather than as a monomer. SUVH2-3xMyc, SUVH9-3xFlag, and DMS3 were co-eluted with the peak at the size between 440 KDa and 669 KDa, which is consistent with the mass spectrometric analysis indicating that both SUVH2 and SUVH9 associated with DMS3 (Figure 1C). The elution peak of MORC6-3xFlag was <440 KDa, indicating that the complex containing MORC6 is smaller than the complex containing SUVH2, SUVH9, or DMS3 (Figure 1C). The small molecular size of the MORC6 elution peak indicated that MORC6 may form a different complex that does not include SUVH2, SUVH9, and DMS3 (Figure 1C).

Mass spectrometric analysis indicated that affinity purification of MORC6-3xFlag produced a large number of peptides corresponding to MORC1 and another MORC family protein, MORC2 (AT4G36280), in *MORC6-3xFlag* transgenic plants (Table 1). Therefore, MORC1, MORC2, and MORC6 may form a tight complex *in vivo*. Affinity purification of MORC6-3xFlag generated three specific silver-stained bands on SDS-PAGE gel (Figure 1D). The three bands were separately cut from the gel and subjected to mass spectrometric analysis. The results indicated the three bands from top to bottom correspond to the MORC6-3xFlag fusion protein, the endogenous MORC6 protein, and the endogenous MORC1 protein (Figure 1D). These results suggest that MORC6 not only forms a homodimer with another MORC6 but also forms a heterodimer with its homologs MORC1 and MORC2 *in vivo*. The formation of the MORC1-MORC6 heterodimer was confirmed by co-IP between MORC1-3xFlag and MORC6-3xMyc in the transgenic plants harboring both *MORC1-3xFlag* and *MORC6-3xMyc* transgenes (Figure 1E). Furthermore, the interaction between MORC6 and MORC1 or MORC2 was examined by yeast two-hybrid assay. The results demonstrated that MORC1, MORC2, and MORC6 can form a homodimer or heterodimer (Figure S2), which is consistent with the result of the gel filtration assay indicating that MORC6 acts in a distinct protein complex *in vivo* (Figure 1C).

**Figure 2:**
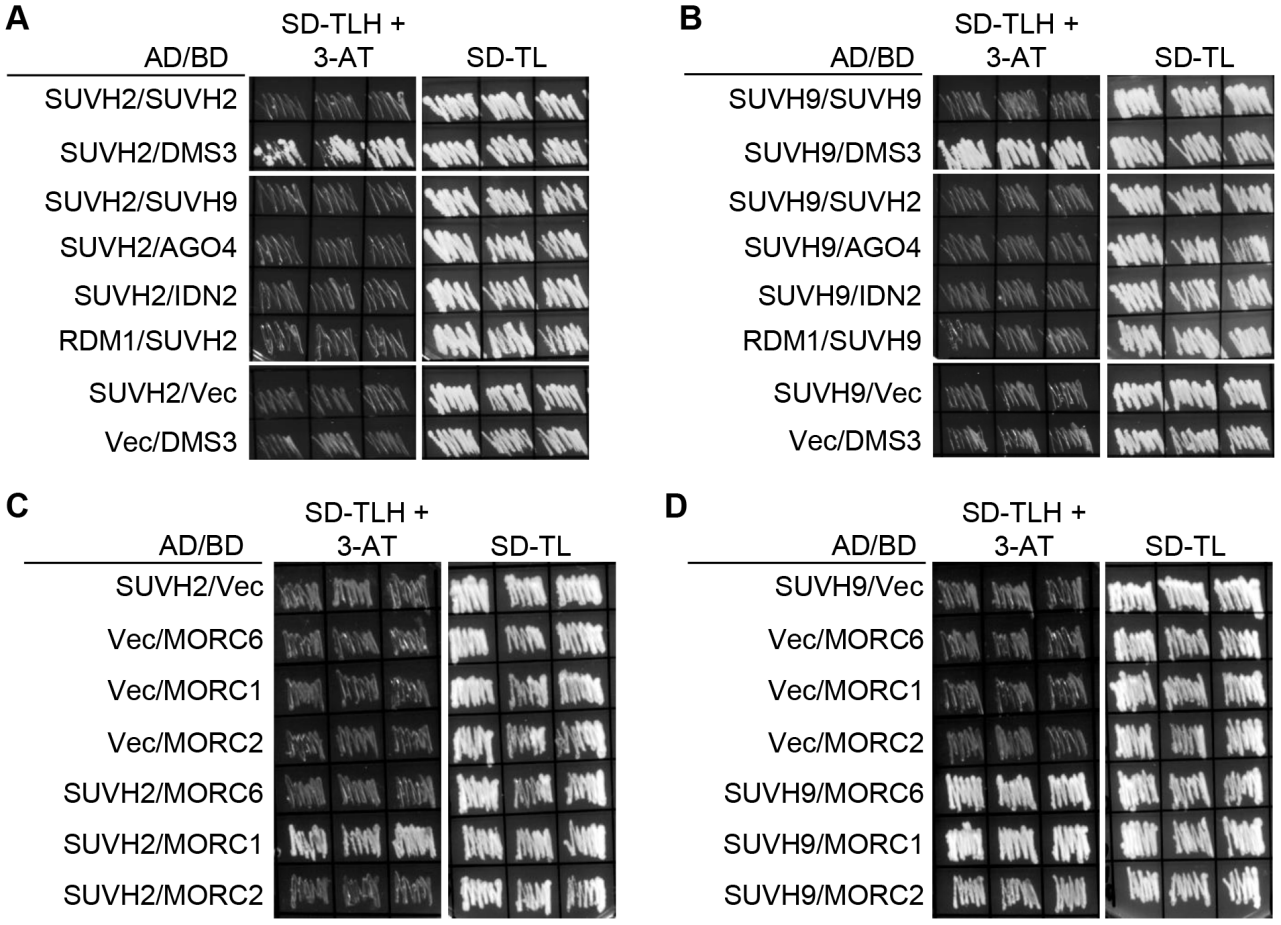
Detection of the interaction of SUVH2 and SUVH9 with RdDM components by yeast two-hybrid assay. The yeast strains harboring *GAL4-AD* and *GAL4-BD* fusion constructs were streaked on plates with indicated media. SD-TLH, the synthetic dropout medium without Trp, Leu, and His. SD-TL, the synthetic dropout medium without Trp and Leu. “Vec” represents the empty pGADT7 or pGBKT7 vector. (A) The interaction of SUVH2 with SUVH9, AGO4, IDN2, and RDM1. (B) The interaction of SUVH9 with SUVH2, DMS3, AGO4, IDN2, and RDM1. (C) The interaction of SUVH2 with the MORC family proteins MORC1, MORC2, and MORC6. (D) The interaction of SUVH9 with the MORC family proteins.

We conducted yeast two-hybrid assay to determine whether SUVH2 and SUVH9 interact with RdDM components and found that both SUVH2 and SUVH9 can interact with DMS3 (Figure 2A, 2B), which is consistent with the results from mass spectrometric analysis (Table 1). Moreover, in yeast two-hybrid assay, SUVH2 weakly interacts with MORC1 rather than with MORC2 and MORC6, whereas SUVH9 interacts with all the three MORC family proteins (Figure 2C, 2D). The interaction of SUVH9 is weaker with MORC2 than with MORC1 and MORC6 (Figure 2D). The interaction between SUVH9 and MORC6 detected by yeast two-hybrid assay is consistent with the results from the affinity purification of both SUVH9-3xFlag and MORC6-3xFlag (Table 1). However, the interaction between SUVH9 and the other two MORC family proteins MORC1 and MORC2 was not found by mass spectrometric assay of SUVH9-3xFlag affinity purification (Table 1). Similarly, the interaction between SUVH2 and MORC1 was also not detected by mass spectrometric assay (Table 1). The failure to detect these interactions is likely due to the low expression levels of these proteins as well as the weak interactions. We constructed truncated SUVH2 and SUVH9 sequences for yeast two-hybrid assay to determine the key domains of SUVH2 and SUVH9 that are required for the interaction with the RdDM components (Figure S3A). Yeast two-hybrid assay indicated that the truncated SUVH2 sequence without its C-terminal SET domain (SUVH2-c) can still interact with DMS3, whereas it cannot interact with MORC1 (Figure S3B). The other truncated versions of SUVH2 (SUVH2-a, SUVH2-b) interact with neither DMS3 nor MORC1 (Figure S3B). Likely, the truncated SUVH9 version without its SET domain (SUVH9-c) can interact with DMS3 but not with MORC1 and MORC6, and the other SUVH9 truncated versions (SUVH9-b, SUVH9-c) can interact with none of the three proteins (Figure S3C). The results suggest that the full length of SUVH2 and SUVH9 is required for their interaction with MORC1 and MORC6, whereas the sequence containing SRA and Pre-SET domains is sufficient for SUVH2 and SUVH9 to interact with DMS3.

### Effect of *suvh2* and *suvh9* on accumulation of Pol IV-dependent siRNAs

**Figure 3:**
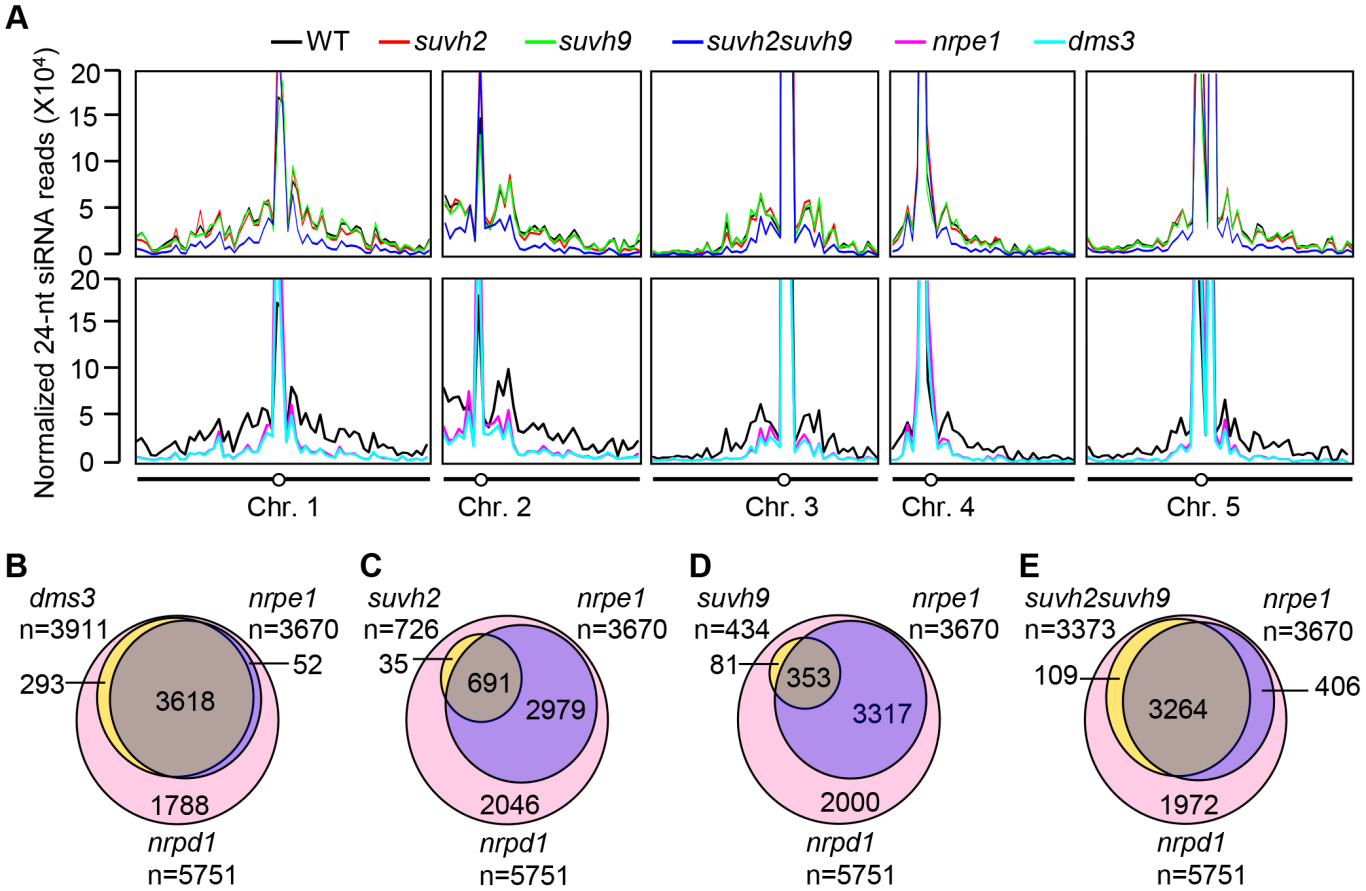
Small RNA analyses by small RNA Northern blotting and small RNA deep sequencing. (A) Diagrams show the distribution of Pol IV-dependent 24-nt siRNAs across all five *Arabidopsis* chromosomes in the wild type, *suvh2*, *suvh9*, *suvh2suvh9*, *nrpe1*, and *dms3*. Normalized 24-nt siRNA reads per ten million in consecutive 500-Kb genomic regions of each chromosome are indicated by the Y coordinate, whereas all five *Arabidopsis* chromosomes are shown along the X coordinate. (B–D) Venn diagrams indicate the numbers and overlaps of siRNA regions that show decreased siRNAs in *dms3* (B), *suvh2* (C), *suvh9* (D), and *suvh2suvh9*. (E) compared to *nrpd1* and *nrpe1*. The results are from the small RNA deep sequencing data for the wild type, *nrpd1*, *nrpe1*, *dms3*, *suvh2*, *suvh9*, and *suvh2suvh9*.

We carried out small RNA deep sequencing to compare the global effect of *suvh2*, *suvh9*, and *suvh2suvh9* relative to that of the canonical RdDM mutants *nrpd1*, *nrpe1*, and *dms3* on the accumulation of Pol IV-dependent siRNAs. We obtained at least 2.6 million uniquely genome-matched small RNA reads for the small RNA libraries of the wild type, *suvh2*, *suvh9*, *suvh2suvh9*, *nrpd1*, *nrpe1*, and *dms3* (Table S1). Pol IV-dependent 24-nt-siRNA regions were defined as the genomic regions in which the abundance of 24-nt siRNAs was significantly reduced in *nrpd1* relative to the wild type. Consistent with previous reports [49]–[51], we found that Pol IV-dependent siRNAs are predominantly enriched in centrometric regions where highly repetitive DNA sequences exist, and are also present in intergenic regions of each chromosome, especially when the regions contain transposable elements (Figure 3A; Table S2). Both NRPE1 and DMS3 are required for the production of Pol V-dependent noncoding RNAs and act at the downstream step of the RdDM pathway [17], [18]. Our small RNA data indicated that NRPE1 and DMS3 predominantly contribute to accumulation of the Pol IV-dependent siRNAs produced from intergenic regions at two arms of each chromosome, but that both proteins have little impact on the Pol IV-dependent siRNAs produced from highly duplicated DNA repeats in centromeric regions (Figure 3A; Table S2), which is consistent with previous reports [50], [51]. Accumulation of Pol IV-dependent siRNAs was only marginally reduced in the *suvh2* and *suvh9* single mutants and the reduction was significantly enhanced in the *suvh2suvh9* double mutant (Figure 3A). The effect of *suvh2suvh9* on accumulation of Pol IV-dependent siRNAs is comparable to that of *nrpe1* and *dms3* at the whole-genome level (Figure 3A), suggesting that SUVH2 and SUVH9 function redundantly at the downstream step of the RdDM pathway.

In total, we identified 5751 siRNA regions where the abundance of 24-nt siRNAs is strongly decreased (>5-fold decrease) in *nrpd1* compared to the wild type (Figure 3B, 3C, 3D, 3E). In 63.8% (3670/5751) of the Pol IV-dependent siRNA regions, 24-nt siRNAs are significantly decreased in *nrpe1* (Figure 3B, 3C, 3D, 3E). Thus, Pol IV-dependent siRNA regions were divided into two groups: Pol V-independent siRNA regions and Pol V-dependent siRNA regions. Of the 3670 Pol V-dependent siRNA regions, 98.6% (3618/3670) have 24-nt siRNAs that are also decreased in *dms3*, whereas only 14.1% (293/2081) of Pol IV-dependent and Pol V-independent siRNA regions are significantly decreased in 24-nt siRNAs in *dms3* (Figure 3B). The highly similar effect of *nrpe1* and *dms3* on 24-nt siRNAs supported the inference that DMS3 facilitates the recruitment of Pol V and mediates the production of Pol V-dependent noncoding RNAs at a later step in the RdDM pathway.

In the *suvh2* and *suvh9* single mutants, 12.6% (726/5751) and 7.5% (434/5751) of the Pol IV-dependent siRNA regions show a significant decrease in 24-nt siRNAs, respectively (Figure 3C, 3D). The low effect of *suvh2* and *suvh9* on Pol IV-dependent siRNAs may account for the functional redundancy between SUVH2 and SUVH9. Although the effect of the *suvh2* and *suvh9* single mutants is quite low, we still find that most of these SUVH2- and SUVH9-dependent siRNA regions (691/726 for SUVH2 and 353/434 for SUVH9) belong to Pol IV- and Pol V-dependent siRNA regions (Figure 3C, 3D). The results suggest that like Pol V, SUVH2 and SUVH9 may act downstream of the primary siRNA biogenesis in the RdDM pathway. In the *suvh2suvh9* double mutant, a high percentage of Pol IV- and Pol V-dependent siRNAs regions (88.9%, 3264/3670) show a decrease in 24-siRNAs, whereas only 5.2% (109/2081) of Pol IV-dependent and Pol V-independent siRNAs regions have decreased 24-nt siRNAs (Figure 3E). The effect of *suvh2suvh9* is comparable to that of the Pol V mutant *nrpe1* and of the DDR complex mutant *dms3* (Figure 3B, 3E)

Based on the small RNA deep sequencing data, we tested the effect of *suvh2* and *suvh9* on well-known Pol IV-dependent siRNAs, which include siRNAs from 180 bp centromeric repeats (*180 bp CEN*), *AtGP1*, *AtSN1*, and *MEA-ISR* loci. As expected, all these Pol IV-dependent siRNAs were blocked in the Pol IV mutant *nrpd1*, whereas *AtSN1* siRNA and *MEA-ISR* siRNA but not *180 bp CEN* siRNA and *AtGP1* siRNA were reduced in the Pol V mutant *nrpe1* and the DDR complex mutant *dms3* (Figure S4). We found that *AtSN1* siRNA and *MEA-ISR* siRNA were weakly reduced in *suvh2* but not in *suvh9*. In the *suvh2suvh9* double mutant, *AtSN1* siRNA and *MEA-ISR* siRNA were reduced to much more extent than that in *suvh2* (Figure S4). The effect of *suvh2suvh9* on accumulation of *AtSN1* siRNA and *MEA-ISR* siRNA is similar to that of *nrpe1* and *dms3* (Figure S4), suggesting that the function of SUVH2 and SUVH9 in Pol V-dependent siRNA accumulation is partially redundant. Neither *suvh2* and *suvh9* single mutants nor *suvh2suvh9* double mutant reduced the Pol V-independent siRNAs, *180 bp CEN* siRNA and *AtGP1* siRNA (Figure S4). These results support the inference that SUVH2 and SUVH9 act together with Pol V and the DDR complex at a later step in the RdDM pathway.

### SUVH2 and SUVH9 are required for Pol V-dependent noncoding RNAs and transcriptional silencing

**Figure 4:**
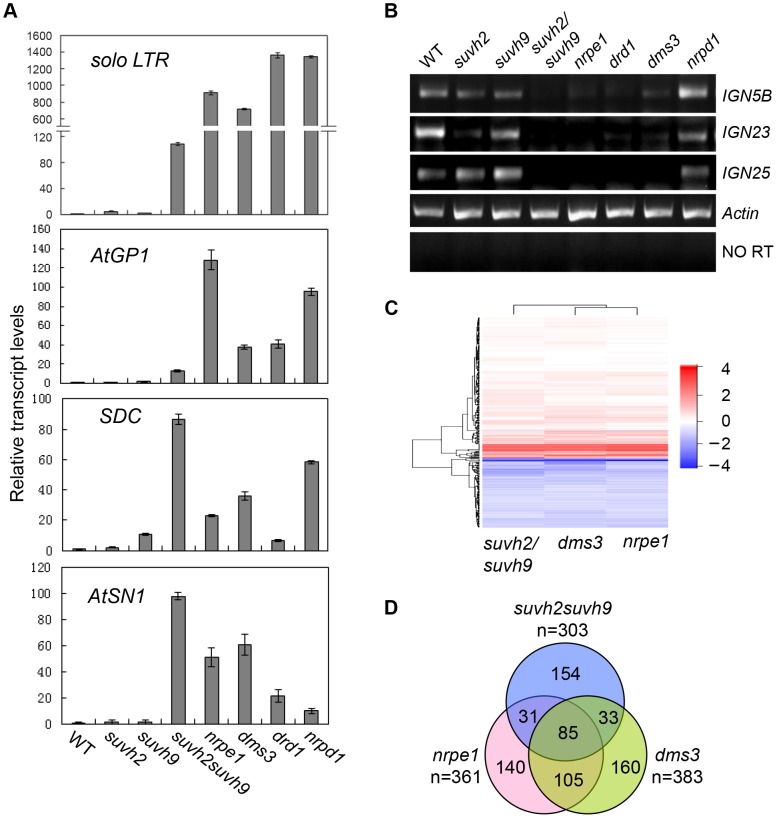
RNA transcript analysis by semi-quantitative RT-PCR and RNA deep sequencing. (A) The effect of *suvh2*, *suvh9*, and *svuh2suvh9* on transcriptional silencing as determined by quantitative RT-PCR. The transcript levels of RdDM loci *solo LTR*, *AtGP1*, *SDC*, *AtSN1* was tested. The actin gene was amplified as an internal control. (B) Pol V-dependent noncoding RNAs from *IGN5B*, *IGN23*, and *IGN25* loci were measured by semiquantitative RT-PCR. No RT indicates the amplification of the actin gene using total RNAs as template without reverse transcription. (C) The genome-wide effect of *suvh2suvh9*, *nrpe1*, and *dms3* on gene expression as determined by RNA deep sequencing. Heat map of log_2_ (Mutant/WT) was shown for each mutant. (D) The numbers and overlaps of the upregulated genes in *suvh2suvh9*, *nrpe1*, and *dms3* relative to the wild type are shown in Venn diagram.

We carried out quantitative RT-PCR to determine the effect of *suvh2* and *suvh9* on transcriptional silencing at RdDM loci. The results indicated that the RNA transcript levels of *solo LTR*, *AtGP1*, *SDC*, and *AtSN1* are markedly induced in the canonical RdDM mutants including *nrpe1*, *drd1*, *dms3*, and *nrpd1* (Figure 4A). The RNA transcript levels of *solo LTR*, *AtGP1*, and *AtSN1* are either not affected or are weakly induced in either the *suvh2* or *suvh9* single mutant (Figure 4A). In the *suvh2suvh9* double mutant, however, the induction of the RNA transcript levels of the three loci are significantly enhanced compared to the levels in the single mutants (Figure 4A). The *SDC* transcript is intermediately induced in *suvh9* rather than in *suvh2* and the transcript is drastically enhanced in the *suvh2suvh9* double mutant (Figure 4A). These results suggest that SUVH2 and SUVH9 function redundantly in transcriptional silencing.

Small RNA analysis indicated that the effect of *suvh2suvh9* on Pol IV-dependent siRNAs is similar to that of the Pol V mutant, *nrpe1* (Figure 3A, 3E). Moreover, both SUVH2 and SUVH9 can interact with DMS3 (Table 1; Figure 1A, 2A, 2B), which acts as a subunit of the DDR complex and is required for Pol V chromatin association at RdDM loci [22], [23]. Therefore, it is possible that SUVH2 and SUVH9 are involved in the accumulation of Pol V-dependent noncoding RNAs. The effect of *suvh2* and *suvh9* on the Pol V-dependent transcript *AtSN1B* was previously noted [48]. We determined the effect of *suvh2*, *suvh9*, and *suvh2suvh9* on the Pol V-dependent noncoding RNAs at *IGN5B*, *IGN23*, and *IGN25* by RT-PCR (Figure 4B; Figure S5). The results showed that the RNA transcript levels of the three tested loci were markedly reduced in *nrpe1*, *drd1*, and *dms3* (Figure 4B; Figure S5), suggesting that the RNA transcripts of these loci are dependent on Pol V. The Pol V-dependent transcripts *IGN5B*, *IGN23*, and *IGN25* were either not affected or marginally reduced in the *suvh2* and *suvh9* single mutants but were markedly reduced in the *suvh2suvh9* double mutant (Figure 4B; Figure S5), which demonstrated that SUVH2 and SUVH9 are required for the production of Pol V-dependent noncoding RNAs at RdDM loci. SUVH2 and SUVH9 are functionally redundant in Pol V-dependent RNA accumulation.

Previous reports suggest that disruption of the RdDM machinery leads to derepression not only of transposable elements but also of their flanking genes [2], [32], [33]. We performed RNA deep sequencing to compare the effect of *suvh2suvh9* vs. *nrpe1* and *dms3* on gene expression at the whole-genome level. For each RNA library, we obtained at least 14.7 million RNA reads that were mapped to the *Arabidopsis* genome (Table S3). The Heat map results indicated that the genome-wide effect of *suvh2suvh9* on gene expression is generally similar to that of *nrpe1* and *dms3* (Figure 4C). The genes upregulated in the RdDM mutants *nrpe1* and *dms3* are thought to be RdDM loci (Table S2). We found that 361, 383, and 303 genes that are significantly (log_2_ (Mutant/WT)>1; P<0.01) upregulated in *nrpe1*, *dms3*, and *suvh2suvh9*, respectively (Table S4; Figure 4D), suggesting that the three mutants have a similar effect on gene expression at the whole-genome level. Of the 383 genes upregulated in *dms3*, 49.6% (190/383) are also upregulated in *nrpe1* (Figure 4D), supporting the inference that DMS3 acts together with NRPE1 in the RdDM pathway. Of the 303 genes upregulated in *suvh2suvh9*, nearly half (49.2%, 149/303) are upregulated in either *nrpe1* or *dms3* (Figure 4D). The data are consistent with the finding that SUVH2 and SUVH9 associate with DMS3 and mediate transcriptional silencing through the RdDM pathway.

### SUVH2 and SUVH9 are involved in the association of Pol V and DMS3 with chromatin at RdDM loci

**Figure 5:**
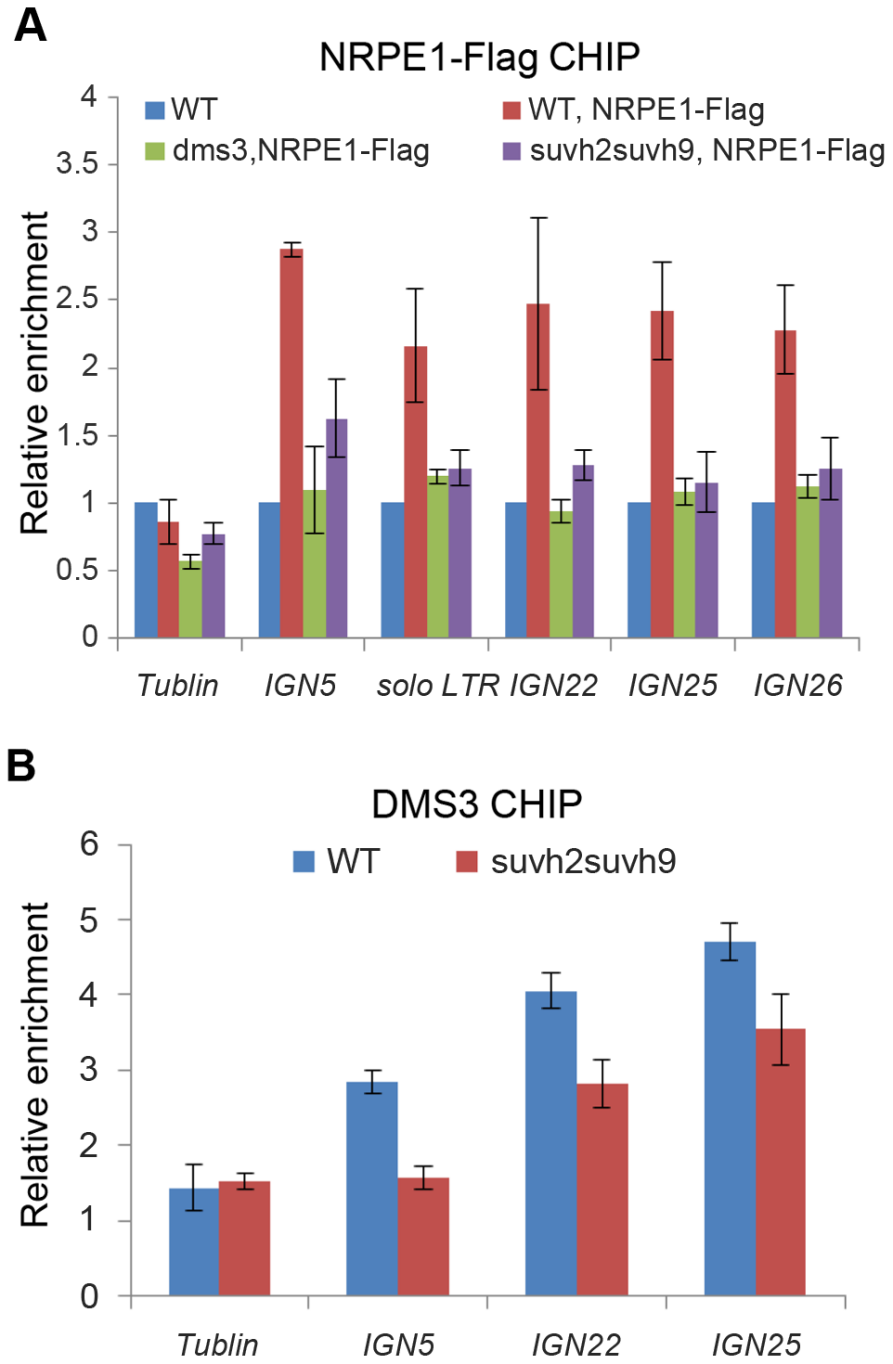
Effect of *suvh2* and *suvh9* on the occupancy of Pol V and the DDR complex at RdDM loci. (A) The effect of *suvh2suvh9* on Pol V occupancy at RdDM loci was determined by CHIP assay with anti-Flag antibody. The enrichment of the largest subunit of Pol V, NRPE1, was measured at *IGN5*, *solo LTR*, *IGN22*, *IDN25*, and *IGN26*. The abundance of NRPE1 on the actin gene was used as an internal negative control. Enrichment of NRPE1-Flag on RdDM loci and the tublin gene was normalized by the abundance of NRPE1-Flag on the actin gene. (B) The effect of *suvh2suvh9* on the occupancy of the DDR complex at RdDM loci was determined by CHIP assay with anti-DMS3 antibody. Enrichment of the DDR complex component DMS3 was determined in the wild type and *suvh2suvh9*. Relative enrichment of DMS3 is shown.

Given that Pol V-dependent noncoding RNAs are markedly decreased in the *suvh2suvh9* double mutant, we determined whether *suvh2suvh9* reduces Pol V chromatin association at RdDM loci. The *NRPE1-Flag* transgene was introduced and expressed in WT, *dms3*, and *suvh2suvh9* equivalently (Figure S6A). By CHIP assay using anti-Flag antibody, we found that NRPE1 was enriched at the RdDM loci including *solo LTR*, *IGN5*, *IGN22*, *IGN25*, and *IGN26*, and that the enrichment was decreased in the DDR complex mutant *dms3* (Figure 5A), which is consistent with previous reports [17], [18]. Interestingly, our results indicated that the enrichment of NRPE1 on chromatin at these RdDM loci was also significantly decreased in *suvh2suvh9* (Figure 5A), suggesting that SUVH2 and SUVH9 are required for Pol V chromatin association. The results demonstrate that SUVH2 and SUVH9 not only physically associate with the DDR complex but also act together with the complex to facilitate Pol V occupancy at RdDM loci.

The DDR complex was previously demonstrated to be co-purified with Pol V [22], whereas no Pol V subunits were identified in SUVH2 and SUVH9 co-purified proteins (Table 1). The results suggest that the role of SUVH2 and SUVH9 in Pol V occupancy is probably indirectly through their action on the DDR complex. Thus, we determined whether *suvh2suvh9* affects the association of the DDR complex with chromatin at RdDM loci. We carried out DMS3 CHIP assay in the wild type as well as in *suvh2suvh9* by using anti-DMS3 antibody. The specificity of the antibody was confirmed by Western blotting assay (Figure S6B). The CHIP assay demonstrated that DMS3 occupancy at RdDM loci including *IGN5*, *IGN22*, and *IGN25* is significantly reduced in *suvh2suvh9* relative to the wild type (Figure 5B), suggesting that SUVH2 and SUVH9 enable the association of the DDR complex with chromatin, thereby facilitating the recruitment of Pol V to RdDM loci.

### SUVH2 and SUVH9 are required for DNA methylation and H3K9me2 at RdDM loci

**Figure 6:**
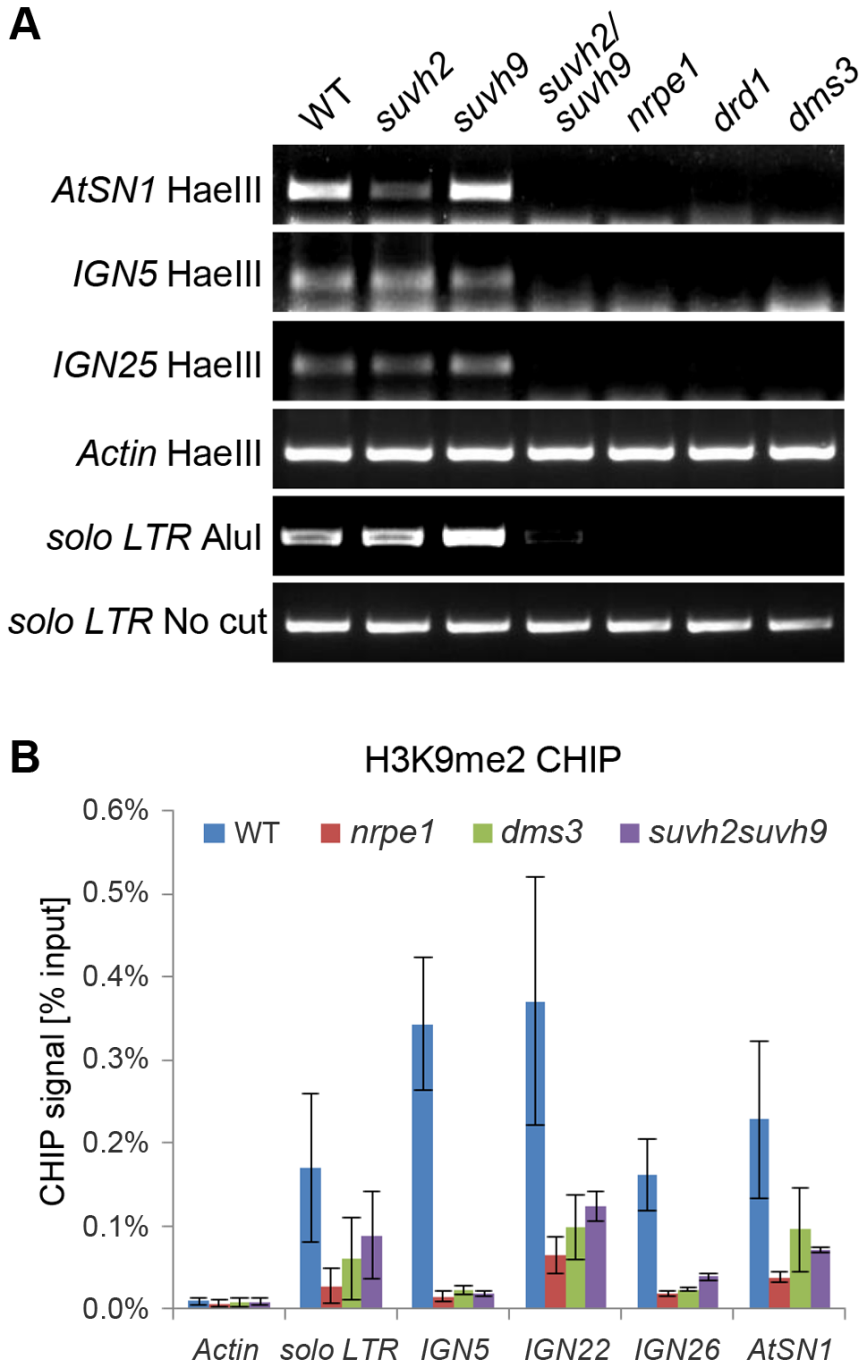
Effect of *suvh2* and *suvh9* on DNA methylation and histone H3K9me2 at RdDM targets. (A) The effect of *suvh2*, *suvh9*, and *suvh2suvh9* on DNA methylation as indicated by chop-PCR. DNA methylation was determined for *AtSN1*, *IGN5*, *IGN25*, and *solo LTR*. Genomic DNA from the wild type, *nrpd1*, *nrpe1*, *dms3*, *suvh2*, *suvh9*, and *suvh2suvh9* was cleaved by the DNA methylation-sensitive restriction enzyme HaeIII or AluI and subjected to semiquantitative PCR. The actin gene, which lacks HaeIII recognition site, was amplified as an internal control. The *solo LTR* locus was amplified using uncut genomic DNA as template for a control. (B) Effect of *suvh2suvh9* on histone H3K9me2 was measured by CHIP assay with anti-H3K9me2 antibody. The RdDM mutants *nrpe1* and *dms3* were included as controls, in which the H3K9me2 levels at indicated RdDM loci were decreased. The actin gene, which lacks detectable H3K9me2, was used as a negative control.

The effect of *suvh2* and *suvh9* on DNA methylation at RdDM loci was evaluated previously [47], [52]. At the genome-wide level, *suvh2* reduces DNA methylation at a large number of RdDM loci, whereas *suvh9* affects only a very small portion of RdDM loci [52], [53]. We determined the effect of *suvh2* and *suvh9* on DNA methylation by chop-PCR (Figure 6A). The results indicated that DNA methylation of *AtSN1*, *IGN5*, *IGN25*, and *solo LTR* was either not reduced or only marginally reduced in the *suvh2* and *suvh9* single mutants but the reduction was greatly enhanced in the *suvh2suvh9* double mutant (Figure 6A). The reduction of DNA methylation in *suvh2suvh9* was comparable to that in the canonical RdDM mutants *nrpe1*, *dms3*, and *drd1* (Figure 6A). The results demonstrate that SUVH2 and SUVH9 are functionally redundant in DNA methylation at RdDM loci.

Previous studies revealed that disruption of canonical RdDM components can lead to reduction of repressive histone H3K9me2 at RdDM loci [17], [45], [46]. To determine whether H3K9me2 at these loci is related to the putative histone methyltransferases SUVH2 and SUVH9, we preformed chromatin immunoprecipitation (CHIP) assay using anti-H3K9me2 antibody. The results indicated that the H3K9me2 levels at *solo LTR*, *IGN5*, *IGN22*, *IGN26*, and *AtSN1* loci are decreased in the Pol V mutant *nrpe1* and the DDR complex mutant *dms3* (Figure 6B), which is consistent with previous results [17], [46]. Moreover, we found that the H3K9me2 levels of these loci are markedly decreased in the *suvh2suvh9* double mutant (Figure 6B), suggesting that SUVH2 and SUVH9 are required for H3K9me2 at RdDM loci.

### The catalytic active site of SUVH2 is dispensable for its function in RdDM and H3K9me2

**Figure 7:**
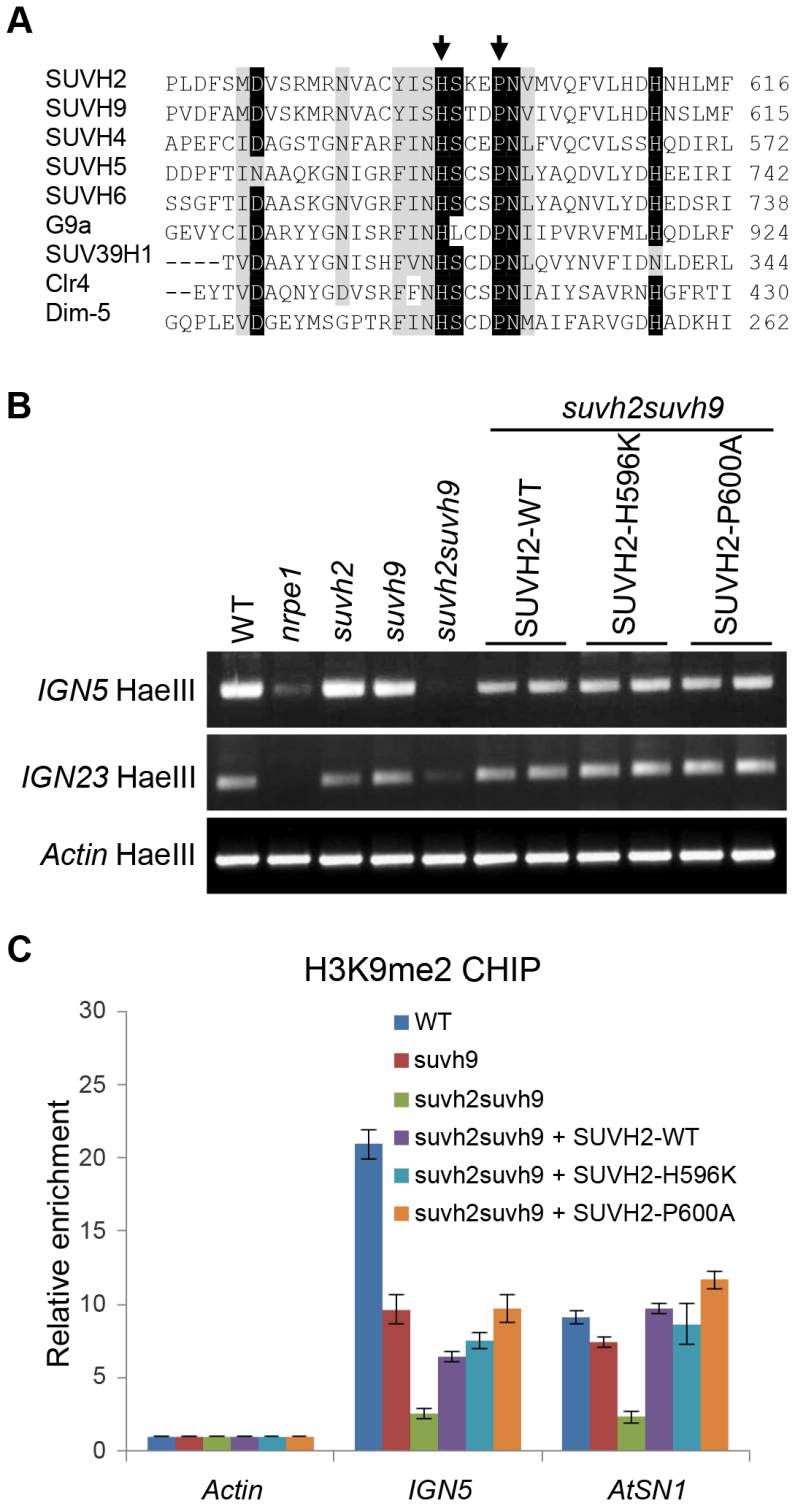
Mutation of the conserved SET domain in SUVH2 has no effect on DNA methylation and H3K9me2 at RdDM loci. (A) Alignment of the catalytic active site in the *Arabidopsis* SET domain proteins SUVH2, SUVH9, SUVH4, SUVH5, and SUVH6, and their homologs including G9a and SUV39H1 in human, Clr4 in fission yeast, and Dim-5 in *Neurospora*. The conserved residues SUVH2-H596 and SUVH2-P600 that were subjected to point mutation are marked with arrows. (B) Site-directed mutagenesis was carried out to introduce the SUVH2-H596K and SUVH2P600A mutations in the SET domain of SUVH2. The construct harboring either wild-type or mutant *SUVH2* sequence was transformed into *suvh2suvh9* for complementation assay. For DNA methylation assay, genomic DNA was digested by the DNA methylation-sensitive restriction enzyme HaeIII followed by PCR. The DNA methylation levels at RdDM loci *IGN5* and *IGN23* were determined. (C) H3K9me2 CHIP assay was performed to test whether the mutant *SUVH2* sequences complement the H3K9me2 defect caused by *suvh2* in *suvh2suvh9* at RdDM loci *IGN5* and *AtSN1*. Enrichment of H3K9me2 was normalized by the abundance of H3K9me2 on the actin gene.

The SET domain is responsible for histone methyltransferase activity and is conserved not only in plants but also in animals and fungi [39]. However, the SET domain-containing proteins SUVH2 and SUVH9 have no detectable histone methyltransferase activity by *in vitro* assay and the overall histone H3K9me2 level is not affected in the *suvh2suvh9* double mutant [47]. It is necessary to determine whether the possible histone methyltransferase activity is required for the function of SUVH2 and SUVH9 in RdDM and H3K9me2 *in vivo*. We mutated two conserved residues (SUVH2-H596K and SUVH2-P600A) in the catalytic active site of the SET domain in SUVH2 and transformed the mutated *SUVH2* sequences into *suvh2suvh9* (Figure 7A). The transgenic plants harboring the stably expressed wild-type or mutant *SUVH2* transgene were used for complementation assay. Previous studies suggest that the two residues SUVH2-H596 and SUVH2-P600 are highly conserved and are essential for the catalytic activity of the SET domain [54], [55]. If histone methyltransferase activity is required for the role of SUVH2 in RdDM and H3K9me2, disruption of the conserved catalytic active site in SUVH2 is expected to block its function *in vivo*. DNA methylation assay demonstrated that the DNA methylation defect in *IGN5* and *IGN23* caused by *suvh2* was complemented by the two mutated *SUVH2* transgene sequences as well as by the wild-type *SUVH2* transgene (Figure 7B). Moreover, H3K9me2 CHIP assay indicated that the two mutations in SUVH2 also have no effect on the in *vivo* function of SUVH2 in H3K9me2 at the RdDM loci *IGN5* and *AtSN1* (Figure 7C). The results suggest that disruption of the putative histone methyltransferase activity of SUVH2 has no effect on the *in vivo* role of SUVH2 in RdDM and histone H3K9me2, supporting the finding that SUVH2 and SUVH9 are not active histone methyltransferases [47]. Histone H3K9me2 at RdDM loci is catalyzed by other histone H3K9 methyltransferases rather than SUVH2 and SUVH9.

## Discussion

**Figure 8:**
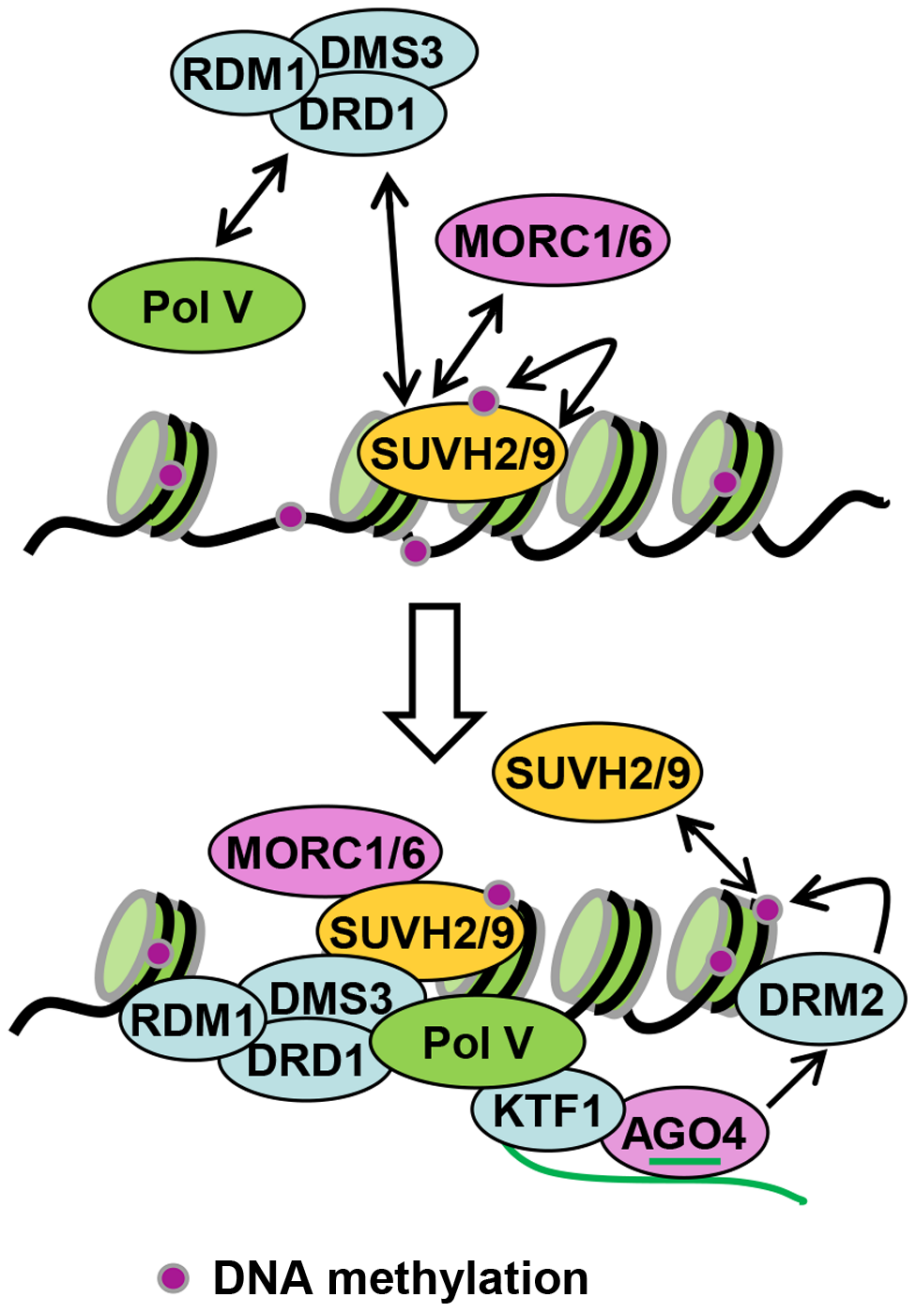
Model for the role of SUVH2, SUVH9 in RdDM. SUVH2 and SUVH9 bind to methylated DNA at RdDM loci and are at least partially required for the occupancy of the DDR complex at the loci. The DDR complex associates with Pol V and facilitates the recruitment of Pol V to RdDM loci. Pol V-produced noncoding RNAs interact with 24nt-siRNAs bound by AGO4, recruiting the *de novo* DNA methyltransferase DRM2 for DNA methylation. The methylated DNA is further bound by SUVH2 and SUVH9, resulting in a self-reinforcing loop that facilitates maintenance of DNA methylation at RdDM loci.

SUVH2 and SUVH9 function redundantly at a downstream step of the RdDM pathway [47], [48]. Previous reports suggest that disruption of the downstream RdDM components including NRPE1 and DMS3 indirectly affects accumulation of Pol IV-dependent 24-nt siRNAs [50], [51]. Our small RNA deep sequencing data indicate that the effect of *suvh2suvh9* on 24-nt siRNA accumulation is comparable to that of *nrpe1* and *dms3* (Figure 3A, 3B, 3E; Figure S4), supporting the functional association of SUVH2 and SUVH9 with the downstream RdDM components NRPE1 and DMS3. We demonstrate that SUVH2 and SUVH9 can also physically associate with the components of the DDR complex and mediate Pol V occupancy at RdDM loci (Table 1; Figure 1, 2, 5A, 6). In the RdDM pathway, Pol V produces long noncoding RNAs that act as scaffold RNAs for the assembly of the RdDM effector complex [17]. The DDR complex is involved in the recruitment of Pol V to chromatin and facilitates the production of Pol V-dependent noncoding RNAs [18], [22], [23]. The association of SUVH2 and SUVH9 with the DDR complex is consistent with the function of SUVH2 and SUVH9 in Pol V occupancy at RdDM loci. The SRA domain of SUVH2 and SUVH9 can bind to methylated DNA [47], but the role of the methylated DNA-binding ability in RdDM remains elusive. Given the finding that SUVH2 and SUVH9 can physically associate with the DDR complex (Table 1; Figure 1, 2), the binding of SUVH2 and SUVH9 to methylated DNA is probably required for the occupancy of the DDR complex on chromatin. Our DMS3 CHIP assay indicates that the occupancy of DMS3 at RdDM sites is significantly reduced in *suvh2suvh9* relative to the wild type (Figure 5B). Thus, reduced Pol V occupancy at RdDM sites in *suvh2suvh9* is likely due to reduced occupancy of DMS3. We propose that the binding of SUVH2 and SUVH9 to methylated DNA mediates the occupancy of the DDR complex at RdDM loci, thereby recruiting Pol V to the loci (Figure 8). Noncoding RNAs produced by Pol V interact with 24-nt siRNAs bound by AGO4 and recruit the *de novo* DNA methyltransferase DRM2 to mediate DNA methylation [17], [18], [21]. Thus, SUVH2 and SUVH9 act as a linker between DNA methylation and Pol V transcription in the RdDM pathway (Figure 8).

Although SUVH2 and SUVH9 have been demonstrated to be required for H3K9me2 at RdDM sites (Figure 6B), SUVH2 and SUVH9 have no detectable histone methyltransferase activity by *in vitro* histone methyltransferase assay [47]. In *Arabidopsis*, another three SUVH proteins SUVH4/KYP, SUVH5, and SUVH6 have histone H3K9 methyltransferase activity and are required for maintenance of histone H3K9 methylation at the whole-genome level [2], [40], [41]. It is possible that H3K9me2 at RdDM sites is catalyzed by these active histone H3K9 methyltransferases. Our results indicate that disruption of the putative catalytic active site of SUVH2 has no effect on the function of SUVH2 in RdDM and H3K9me2 (Figure 7A, 7B, 7C), supporting the inference that SUVH2 and SUVH9 act as adaptor proteins that are involved in Pol V recruitment but not as active histone methyltransferases. H3K9me2 of RdDM loci requires not only SUVH2 and SUVH9 but also other RdDM components including NRPD1, NRPE1, and AGO4 [17], [45], [46], suggesting that SUVH2 and SUVH9 are implicated in H3K9me2 through the RdDM pathway.

SUVH2 and/or SUVH9 can associate not only with the DDR complex but also with the MORC family proteins (Table 1; Figure 1, 2). A recent report suggests that involvement of MORC1 and MORC6/DMS11 in transcriptional silencing is not related to DNA methylation [36], whereas another two reports indicate that MORC6/DMS11 is a new component of the RdDM machinery [37], [38]. Our results demonstrate that MORC6/DMS11 and its homologs MORC1 and MORC2 associate with SUVH9 (Table 1; Figure 1B, 1C, 2D), which is required for Pol V occupancy in the RdDM pathway (Figure 5A). MORC6/DMS11 was previously demonstrated to be involved in the production of Pol V-dependent RNAs [37]. Our results suggest that involvement of MORC6/DMS11 in the production of Pol V-dependent RNAs is related to the physical association of SUVH2 and SUVH9 with MORC6/DMS11. SUVH2, SUVH9, the DDR complex, and the MORC family proteins act together and mediate Pol V occupancy at RdDM loci, thereby facilitating the production of Pol V-dependent noncoding RNAs (Figure 8). Moreover, the MORC family proteins can also mediate the change of chromatin superstructure and lead to transcriptional silencing without involvement of DNA methylation [36]. Our mass spectrometric assay has identified CHB3/SWI3D, a subunit of the SWI/SNF-type chromatin-remodeling complex, as a MORC6-associating protein in affinity purification of MORC6-3xFlag (Table 1). The SWI/SNF-type chromatin-remodeling complex was recently demonstrated to act in Pol V-dependent noncoding RNA-mediated transcriptional silencing by establishing positioned nucleosomes on specific genomic loci [33]. Therefore, in addition to the role of the MORC family proteins in the RdDM pathway, they may also act with the SWI/SNF-type chromatin-remodeling complex to contribute to RNA-mediated transcriptional silencing through a DNA methylation-independent manner.

Our results demonstrate that MORC1, MORC2, and MORC6 can form a homodimer or a heterodimer *in vivo* (Table 1; Figure 1D, 1E; Figure S2). A recent report suggests that the GHLK ATPase domain-containing MORC6/DMS11 can physically interact with the SMC hinge domain-containing protein DMS3 and form a functional analog of an authentic SMC protein, which functions in a dimer and binds complementary DNA strands during sister chromatid cohesion, chromatin condensation, and DNA repair [37], [56]. Given that DMS3 can form a homodimer as described previously [20], a MORC dimer and a DMS3 dimer may form a functional SMC analog in the RdDM pathway, which may help produce Pol V-dependent noncoding RNAs. However, no DMS3 peptide is identified by affinity purification of MORC6-3xFlag (Table 1), which does not support the tight *in vivo* interaction between the MORC dimer and the DMS3 dimer.

Overall, our results indicate that SUVH2 and SUVH9 are physically and functionally associated with the DDR complex and the MORC family proteins and facilitate Pol V occupancy at RdDM loci, concluding that SUVH2 and SUVH9 are canonical components of the RdDM machinery. SUVH2 and SUVH9 may bind to methylated DNA at RdDM loci and interact with the DDR complex at the loci, thereby recruiting Pol V to produce noncoding RNAs. Moreover, we demonstrate that the putative histone methyltransferase activity of SUVH2 is dispensable for the function of SUVH2 in RdDM and H3K9me2 at RdDM loci. Involvement of SUVH2 in H3K9me2 at RdDM loci is via its action in the RdDM pathway. Further study is required to elucidate how SUVH2, SUVH9, the DDR complex, and the MORC family proteins are coordinated to mediate Pol V occupancy at RdDM loci.

## Materials and Methods

### Plant materials, cloning, and generation of transgenic plants

The *Arabidopsis* materials included the wild-type Col-0, *suvh2* (Gabi_kat_516A07), *suvh9* (SALK_048033), *suvh2suvh9* [48], *nrpd1-3* (SALK_128428), *nrpe1-11* (SALK_029919C), *dms3* (Salk_125019C), *drd1-6* [19], *morc1* (Salk_008610), and *morc6* (GK-599B06-023140). The seedlings were grown on MS medium plates under long-day conditions (16 h day time and 8 h night time) at 22°C. Two-week-old seedlings were directly used for the study or transplanted into soil when adult plants or flower tissues were needed.

The *Flag-* and/or *Myc-*tagged *SUVH2*, *SUVH9*, *MORC1*, and *MORC6* genomic sequences were constructed into the modified binary pCAMBIA1305 vector or pRI909 vector. The DNA primers used for constructing were listed in Table S5. In these constructs, the genes were driven by their own native promoters, and the tags were fused to the C-terminal of the corresponding proteins. The constructs were transformed to wild-type plants by agrobacteria infection. T1 transgenic plants were grown on MS medium plates supplemented with 20 mg/ml hygromycin or 50 mg/ml kanamycin. The resistant seedlings were transplanted into soil and grown for the study. For complementation assay, the construct harboring the native promoter-driven *SUVH2-3xMyc* sequence was transformed into *suvh2suvh9*. The wild-type *SUVH2* genomic sequence was subjected to site-directed mutagenesis, introducing the SUVH2-H596K and SUVH2-P600A mutations in the *SUVH2-3xMyc* construct.

### Affinity purification and mass spectrometric analysis

A 3-g quantity of flower tissue from *SUVH2-3xMyc*, *SUVH9-3xFlag*, or *MORC6-3xFlag* transgenic plants was harvested for affinity purification by anti-Flag antibody. Flower tissue was ground in liquid nitrogen and suspended in Lysis buffer (50 mM Tris [pH 7.6], 150 mM NaCl, 5 mM MgCl2, 10% glycerol, 0.1% NP-40, 0.5 mM DTT, 1 mM PMSF, and 1 protease inhibitor cocktail tablet/50 ml [Roche]). The final supernatant was incubated with Anti-Flag M1 agarose (Sigma, A 4596) in Lysis buffer at 4°C for 2.5 h and then precipitated by centrifugation. The precipitant was washed four times, and the agarose-bound proteins were eluted with 3xFlag peptide (Sigma, F 4799).

The eluted proteins were run on a 10–12% SDS-PAGE gel and then subjected to silver staining with the ProteoSilver Silver Stain Kit (Sigma, PROT-SIL1). Total eluted proteins or specific target bands were cut from gels and extracted. Mass spectrometric analysis was performed as described previously [30]. Briefly, the peptides were eluted on a capillary column (50 µm×10 cm) packed with 5-µm spherical C18 reversed-phase material (YMC, Kyoyo, Japan). The eluted peptides were loaded onto a LTQ mass spectrometer (Thermo Fisher Scientific) for analysis. The peptide sequence data were searched against the IPI (International Protein Index) *Arabidopsis* protein database on the Mascot server (Matrix Science Ltd., London, UK).

### Co-immunoprecipitation and gel filtration

*SUVH2-3xMyc* and *SUVH9-3xFlag* transgenic plants were used to determine whether SUVH2 and SUVH9 interact with DMS3 by co-immunoprecipitation. *SUVH9-3xFlag* transgenic plants were crossed to *MORC6-3xMyc* transgenic plants, and the offspring plants harboring both transgenes were used to determine whether SUVH9 interact with MORC6. For co-IP, protein extracts were incubated with the agarose-conjugated antibodies in Lysis buffer at 4°C for 2–3 h and precipitated by centrifugation. The precipitant was washed four times and then boiled for 5 min in 1×SDS sample buffer. After centrifugation, the supernatant was run on an SDS-PAGE gel, followed by Western blotting.

For gel filtration, total protein extracts from wild-type or transgenic plants were loaded onto a Superose 6 10/300 GL column (GE Healthcare, 17-5172-01) and the eluted solution was harvested once per 500 µl. The eluted fractions were run on a 10–12% SDS-PAGE gel and were then subjected to Western blotting. Standard proteins were used to determine the sizes of the eluted fractions.

### Yeast two-hybrid assay

The cDNA sequences were cloned into pGADT7 and pGBKT7 vectors in frame to the C-termini of *GAL4-AD* and *GAL4-BD*, respectively. The DNA primers for cloning were listed in Table S5. The yeast strain PJ694a was co-transformed with pGADT7 and pGBKT7 constructs and grown on the synthetic dropout medium minus Trp and Leu (SD-TL). The positive yeast colonies were streaked on both SD-TL and SD-TLH (the synthetic dropout medium minus Trp, Leu, and His) for growth assay. Growth of transformed positive yeast strains on SD-TLH indicates the interaction between GAL-AD fusion protein and GAL4-BD fusion protein in corresponding yeast strains. 20 mM 3-AT was used to inhibit the background growth of transformed strains on SD-TLH medium.

### Small RNA deep sequencing

Small RNA was extracted from two-week-old seedlings as described previously [57] and subjected to Illumina sequencing. Raw reads from small RNA libraries were processed to remove adapter sequences and were classified according to the barcodes. Small RNAs with 18 to 27 nt were mapped to the *Arabidopsis* genome (TAIR10) using the Bowtie program [58], and only perfectly matched reads were extracted for subsequent analysis. The whole *Arabidopsis* genome was split into 500-bp bins, and the appearances of 24-nt reads in each bin were counted. The read counts were then normalized by the library size. Reads per ten million (RPTM) were calculated for the adjusted small RNA abundance in each bin. The bins with low reads (<200 RPTM in all samples) were removed, and the regions were referred to as Pol IV-dependent siRNA regions if the RPTM value was five-time greater in the wild type than in the Pol IV mutant *nrpd1*. We also determine the RPTM value in Pol IV-dependent siRNA regions using 500-Kb windows to plot the profile of Pol IV-dependent siRNAs across chromosomes.

### RNA deep sequencing-based gene expression analysis

The mRNA extracted from each genotype was used for Illumina library construction. The libraries were subjected to single-end Illumina sequencing. For data analysis, 45-bp sequences were mapped to the *Arabidopsis* genome (TAIR10) using TopHat [59]. Only reads mapped uniquely to the genome with a maximum of two mismatches were used for further analysis. Cufflinks [60] was used to quantify changes in gene expression between genotypes. Genes with a significant differential expression (P<0.05) was chosen to plot Heat map using Gplots package in R.

### Analyses of RNA transcripts by RT-PCR

Total RNA was extracted by the routine method with Trizol as described previously [57]. For RT-PCR, total RNA was treated with DNase to remove residual DNA contamination in each RNA sample. RT-PCR was performed with the TAKARA one-step RT-PCR kit. Either sequence-specific primers or oligo-dT were used for reverse transcription. The RNA transcript levels were determined by semi-quantitative RT-PCR or quantitative RT-PCR. All RT-PCR results were biologically repeated at least two times. The primers used for RT-PCR were listed in Table S5.

### DNA methylation analysis

DNA methylation was tested by chop-PCR. Genomic DNA was cleaved with the methylation-sensitive restriction enzyme HaeIII or AluI, followed by PCR. The abundance of PCR products indicates the DNA methylation levels of the amplified loci. The actin gene sequence, which lacks an HaeIII recognition site, was amplified as an internal control. The DNA methylation analyses were repeated at least two times.

### CHIP assay

The levels of histone H3K9me2, NRPE1-Flag and DMS3 on chromatin were determined by CHIP assay as described previously [17]. Two-week-old seedlings were soaked in 0.5% formaldehyde under vacuum for cross linking. After chromatin was extracted and sonicated, it was immunoprecipitated with agarose-conjugated antibodies. Anti-Flag M2 Affinity Gel (A2220, Sigma) is directly used for immunoprecipitation, whereas anti-H3K9me2 antibody (ab1220, Abcam) and anti-DMS3 antibody [30] are conjugated with Protein A agarose followed by immunoprecipitation. Precipitated DNA as well as input DNA was subjected to quantitative PCR. The actin gene that has no NRPE1 and H3K9me2 enrichment was used as an internal control. Enrichment of NRPE1-Flag, H3K9me2 and DMS3 on chromatin was normalized by that on the actin gene. The primers used for quantitative PCR were listed in Table S5. The indicated results were obtained from three independent replicates.

## Supporting Information

Figure S1 Detection of the interaction between the histone methyltransferases and the RdDM component DMS3 by co-IP. (A) Detection of the interaction between SUVH2 and DMS3. The protein extracts from *SUVH2-Myc* transgenic plants were precipitated using anti-Myc antibody and the precipitates were subjected to Western blotting. The band labeled “*” is an unspecific band. (B, C) Detection of the interaction between SUVH9 and DMS3. The protein extracts from *SUVH9-Flag* transgenic plants were precipitated using either anti-Flag antibody (B) or anti-DMS3 antibody (C), and the precipitates were subjected to Western blotting.
(PDF)

Figure S2 The interaction of MORC6 with MORC1 and MORC2 was determined by yeast two-hybrid assay. MORC1, MORC2, and MORC6 were separately cloned into pGADT7 and/or pGBKT7 vectors and transformed into the yeast strain PJ694a for yeast two-hybrid assay.
(PDF)

Figure S3 Detection of the interaction between truncated SUVH2 and SUVH9 sequences and RdDM components by yeast two-hybrid assay. Three individual yeast strains harboring the indicated GAL4-AD and GAL4-BD fusion constructs were streaked on the yeast synthetic dropout medium minus Trp, Leu, and His (SD-TLH) but supplemented with 3-AT and on the SD medium minus Trp and Leu (SD-TL). (A) Diagram of the truncated SUVH2 and SUVH9 sequences used in yeast two-hybrid assay. (B) The interaction between truncated SUVH2 sequences and the RdDM components DMS3 and MORC1. (C) The interaction between truncated SUVH9 sequences and the RdDM components DMS3, MORC6, and MORC1.
(PDF)

Figure S4 Accumulation of Pol IV-dependent 24-nt siRNAs as determined by small RNA deep sequencing. Pol IV-dependent 24-nt siRNA reads from 180 bp CEN, *AtGP1*, *AtSN1*, and *MEA-ISR* loci were counted based on small RNA deep sequencing data. The numbers of ta-siRNA255 reads in each corresponding library were used as controls to normalize the Pol IV-dependent siRNA reads. Shown are relative accumulation levels of indicated siRNAs in the wild type as well as in each mutant.
(PDF)

Figure S5 The Pol V-dependent RNA transcripts *IGN5B*, *IGN23*, and *IGN25* were examined by quantitative RT-PCR. The mutants *nrpe1*, *drd1*, and *dms3*, which were previously demonstrated to be required for Pol V-dependent RNA transcripts, were included as controls. The *nrpd1* mutant that has no effect on Pol V-dependent RNA transcripts was also used as a control.
(PDF)

Figure S6 Determination of the *NRPE1-3xFlag* and *DMS3* expression levels by Western blotting. (A) The *NRPE1-3xFlag* transgene was introduced into the wild type, *dms3*, and *suvh2suvh9*, respectively. The *NRPE1-3xFlag* expression was determined in transgenic plants as well as in the wild-type control by using anti-Flag antibody. The Ponceau S-stained rubisco protein is shown as a loading control. (B) The *DMS3* expression level was determined in the wild type, *dms3*, and *suvh2suvh9* by using anti-DMS3 antibody.
(PDF)

Table S1 Summary of small RNA deep sequencing data.
(XLS)

Table S2 Numbers of Pol IV-dependent siRNA reads in 500-bp windows of the *Arabidopsis* genome in each ecotype.
(XLS)

Table S3 Summary of RNA deep sequencing data.
(XLS)

Table S4 List of differentially expressed genes in *nrpe1*, *dms3*, and *suvh2suvh9* detected by RNA deep sequencing.
(XLS)

Table S5 List of DNA oligonucleotides used in this study.
(XLS)
