# Supplementary figures and images for: The SET Domain Proteins SUVH2 and SUVH9 Are Required for Pol V Occupancy at RNA-Directed DNA Methylation Loci
Source: PLoS Genet. 2014 Jan 22;10(1):e1003948. doi: 10.1371/journal.pgen.1003948 (PMC3898904; doi:10.1371/journal.pgen.1003948)

Supplemental Figure S1

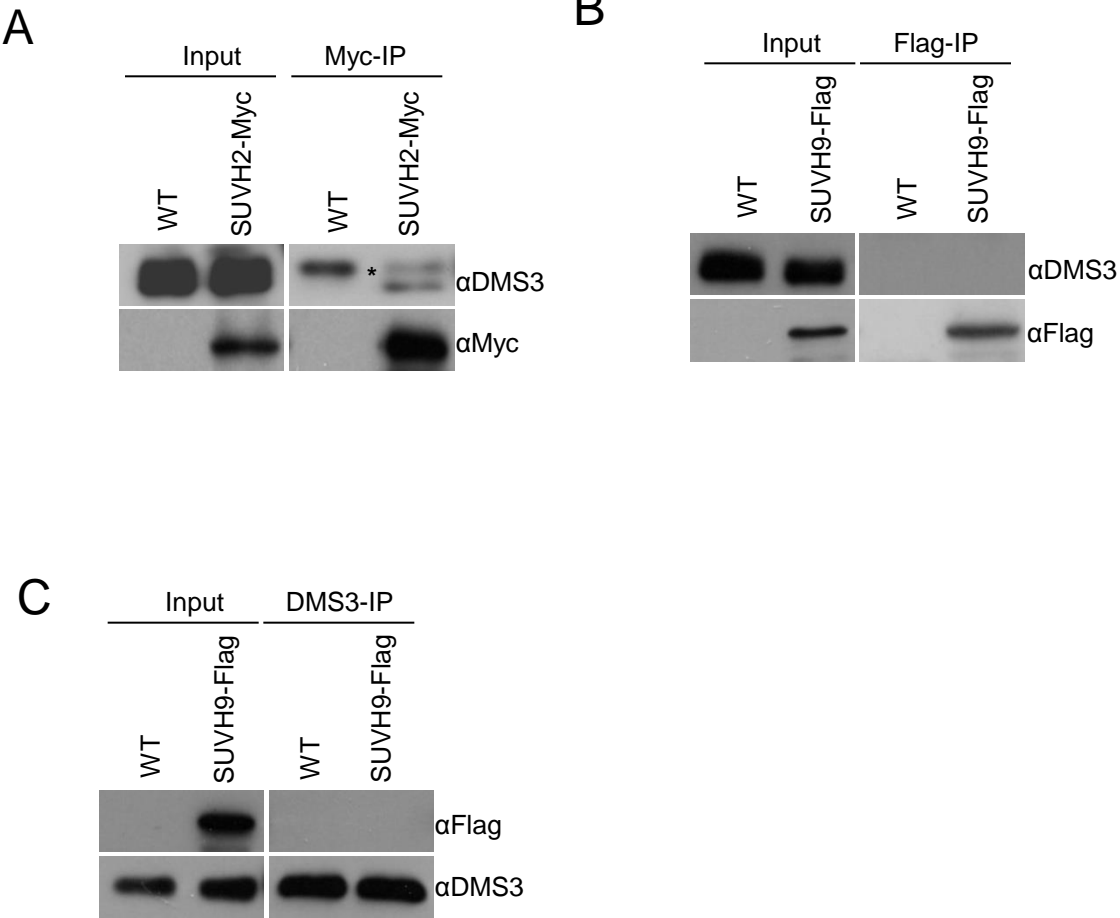

Supplement: Figure S1 — Detection of the interaction between the histone methyltransferases and the RdDM component DMS3 by co-IP. (A) Detection of the interaction between SUVH2 and DMS3. The protein extracts from SUVH2-Myc transgenic plants were precipitated using anti-Myc antibody and the precipitates were subjected to Western blotting. The band labeled “*” is an unspecific band. (B, C) Detection of the interaction between SUVH9 and DMS3. The protein extracts from SUVH9-Flag transgenic plants were precipitated using either anti-Flag antibody (B) or anti-DMS3 antibody (C), and the precipitates were subjected to Western blotting. (PDF) [file pgen.1003948.s001.pdf]

Supplemental Figure S2

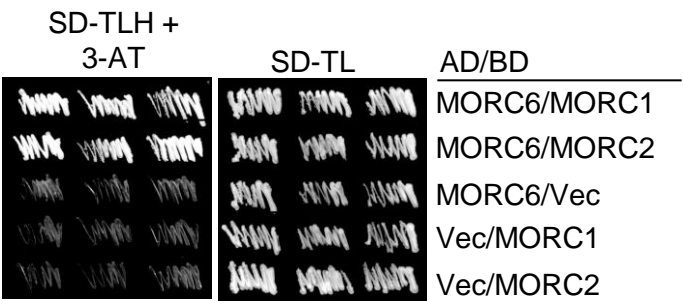

Supplement: Figure S2 — The interaction of MORC6 with MORC1 and MORC2 was determined by yeast two-hybrid assay. MORC1, MORC2, and MORC6 were separately cloned into pGADT7 and/or pGBKT7 vectors and transformed into the yeast strain PJ694a for yeast two-hybrid assay. (PDF) [file pgen.1003948.s002.pdf]

Supplemental Figure S3

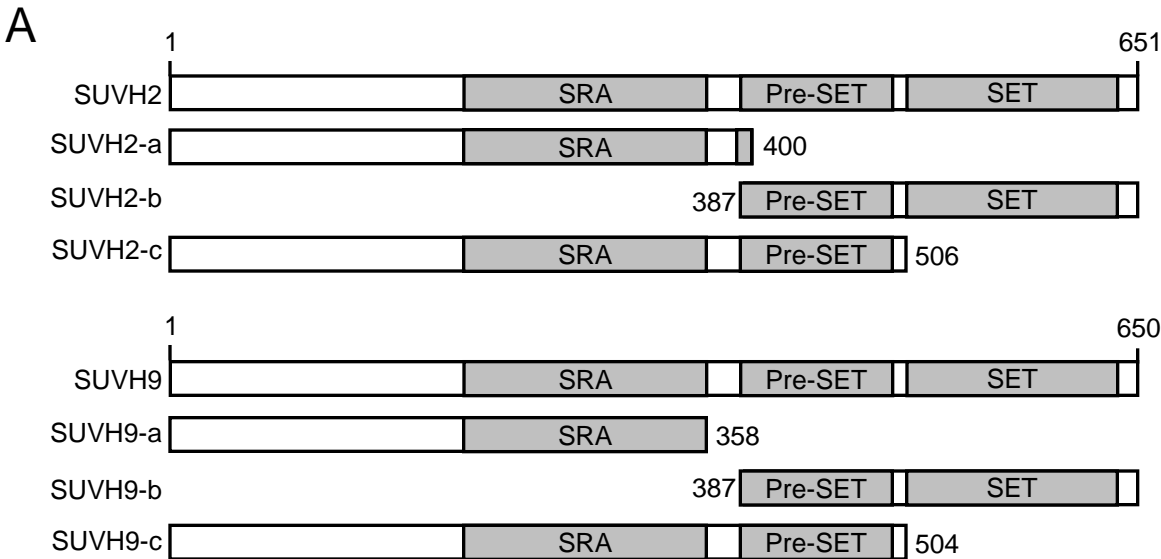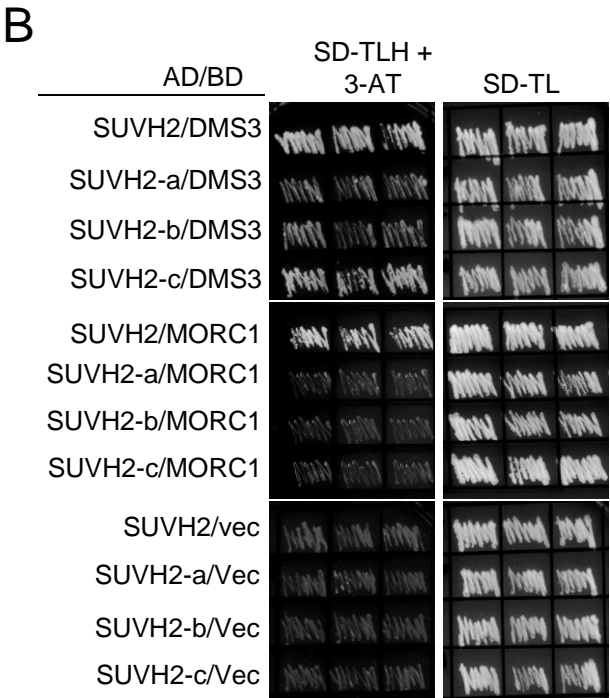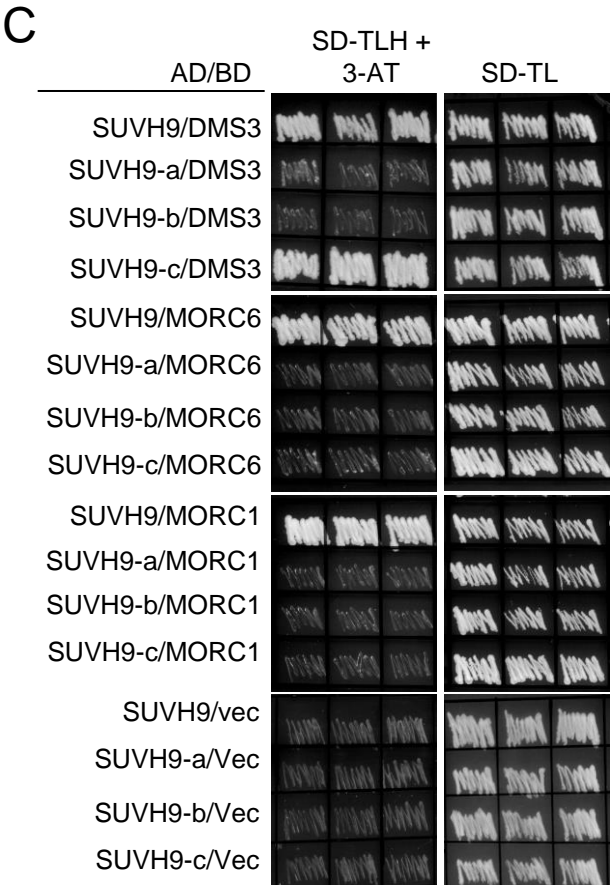

Supplement: Figure S3 — Detection of the interaction between truncated SUVH2 and SUVH9 sequences and RdDM components by yeast two-hybrid assay. Three individual yeast strains harboring the indicated GAL4-AD and GAL4-BD fusion constructs were streaked on the yeast synthetic dropout medium minus Trp, Leu, and His (SD-TLH) but supplemented with 3-AT and on the SD medium minus Trp and Leu (SD-TL). (A) Diagram of the truncated SUVH2 and SUVH9 sequences used in yeast two-hybrid assay. (B) The interaction between truncated SUVH2 sequences and the RdDM components DMS3 and MORC1. (C) The interaction between truncated SUVH9 sequences and the RdDM components DMS3, MORC6, and MORC1. (PDF) [file pgen.1003948.s003.pdf]

# Supplemental Figure S4

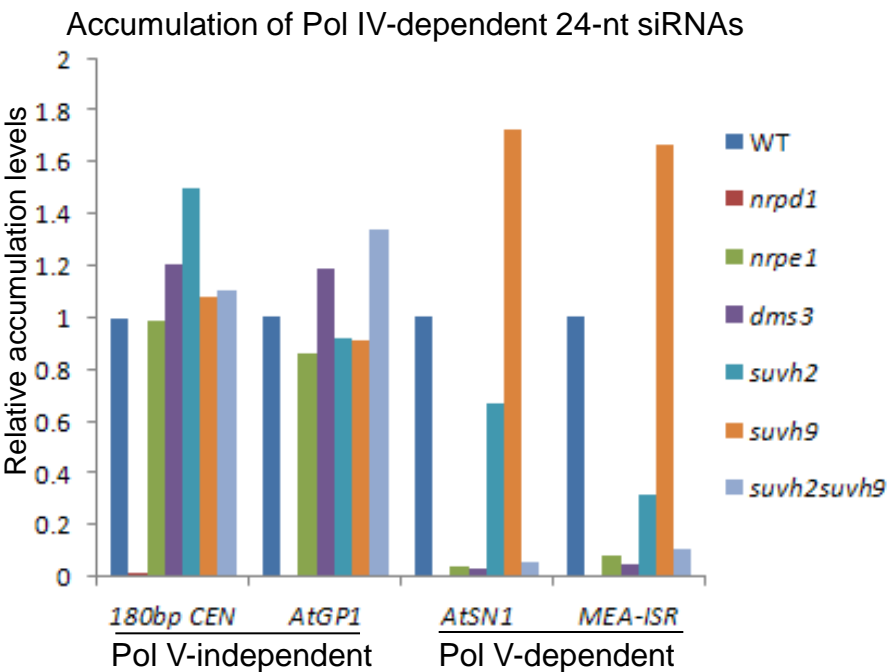

Supplement: Figure S4 — Accumulation of Pol IV-dependent 24-nt siRNAs as determined by small RNA deep sequencing. Pol IV-dependent 24-nt siRNA reads from 180 bp CEN, AtGP1, AtSN1, and MEA-ISR loci were counted based on small RNA deep sequencing data. The numbers of ta-siRNA255 reads in each corresponding library were used as controls to normalize the Pol IV-dependent siRNA reads. Shown are relative accumulation levels of indicated siRNAs in the wild type as well as in each mutant. (PDF) [file pgen.1003948.s004.pdf]

Supplemental Figure S5

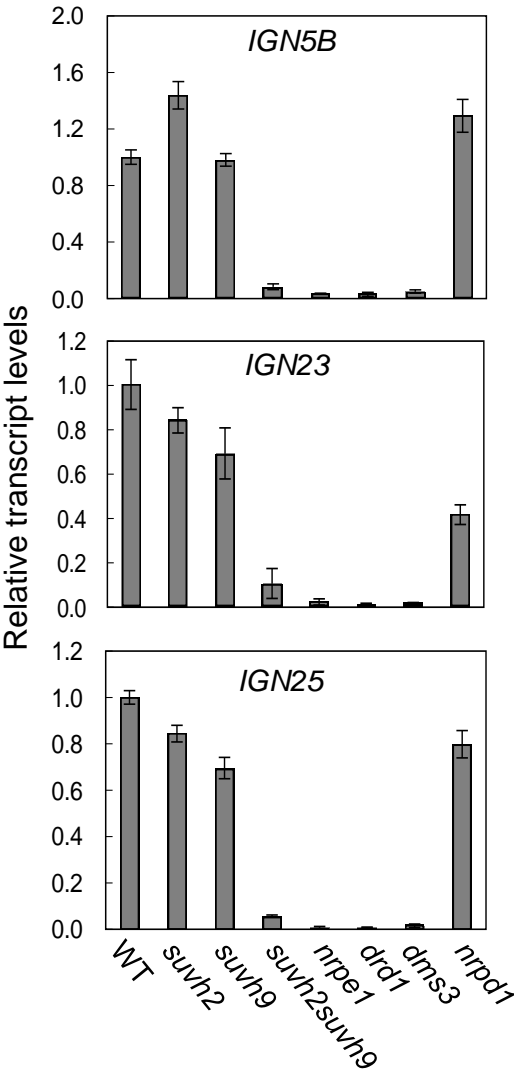

Supplement: Figure S5 — The Pol V-dependent RNA transcripts IGN5B, IGN23, and IGN25 were examined by quantitative RT-PCR. The mutants nrpe1, drd1, and dms3, which were previously demonstrated to be required for Pol V-dependent RNA transcripts, were included as controls. The nrpd1 mutant that has no effect on Pol V-dependent RNA transcripts was also used as a control. (PDF) [file pgen.1003948.s005.pdf]

Supplemental Figure S6

A

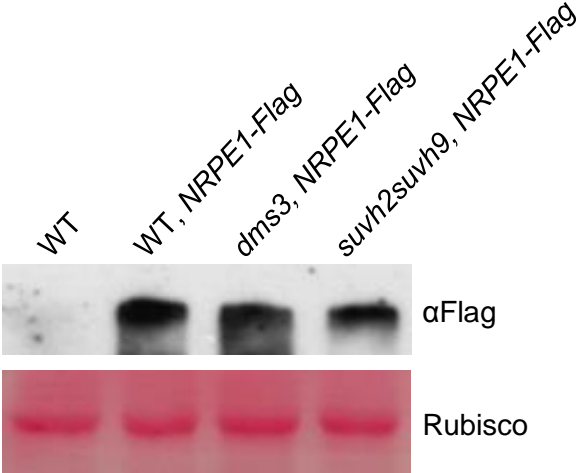

B

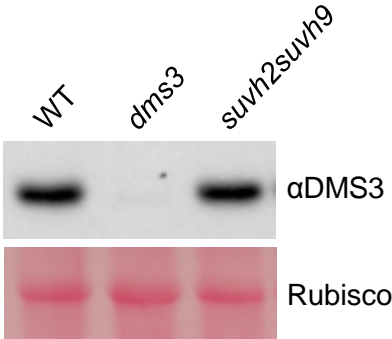

Supplement: Figure S6 — Determination of the NRPE1-3xFlag and DMS3 expression levels by Western blotting. (A) The NRPE1-3xFlag transgene was introduced into the wild type, dms3, and suvh2suvh9, respectively. The NRPE1-3xFlag expression was determined in transgenic plants as well as in the wild-type control by using anti-Flag antibody. The Ponceau S-stained rubisco protein is shown as a loading control. (B) The DMS3 expression level was determined in the wild type, dms3, and suvh2suvh9 by using anti-DMS3 antibody. (PDF) [file pgen.1003948.s006.pdf]
